# Design for Health: Human-Centered Design Looks to the Future

**DOI:** 10.9745/GHSP-D-21-00608

**Published:** 2021-11-29

**Authors:** Tracy Johnson, Shilpa Das, Nikki Tyler

**Affiliations:** a Bill & Melinda Gates Foundation, Seattle, WA, USA.; b National Institute of Design, Ahmedabad, India.; c United States Agency for International Development, Washington, DC, USA.

## Abstract

Global health practitioners and designers recognize that real questions remain around the application of human-centered design in global health. This supplement seeks to clarify its value and document lessons learned, while also distilling and demystifying design. We hope the supplement can act as an inspiration in building a shared vision of how design can advance impact in global health.

## INTRODUCTION

Before the coronavirus disease (COVID-19) pandemic, the global community had been cautiously approaching a new era in global health. Between 1990 and 2015, maternal mortality worldwide dropped by 44%.^1^ Since 2000, the global under-5 mortality rate declined by 44%, new HIV cases decreased by 35%,^2^ and the incidence rate of TB declined by 19%. However, even with this progress, the world had not been on track to achieve its Sustainable Development Goal targets, with inequity increasing.^3^

While the ripple effects of COVID-19 will take years, if not decades, to untangle, early data demonstrate its stark impact. As of April 2021, antenatal care visits fell by 43%, malaria diagnosis fell by 31%, and HIV testing dropped 41%.^4^ COVID-19 has also highlighted the extent to which, even in the face of progress, longstanding societal inequities remain intact.^5^^,^^6^

COVID-19 has given global health practitioners yet another opportunity to radically rethink how we work and engage in global health moving forward. Trends like demographics, urbanization, slower and unequal economic growth, and climate change, all pose huge challenges. Our global health goals depend on our collective efforts to problem solve, strategically take risks, and quickly iterate/adapt to spur more impactful solutions.

Global health practitioners and designers alike recognize that real questions remain about the application and complementarity of design in global health.* Design for Health—jointly led by the Bill & Melinda Gates Foundation and the U.S. Agency for International Development— brought together donors, designers, researchers, implementing partners, and country governments to explore these questions more fully. Members of this community came together in this special issue of *Global Health: Science and Practice* to build upon lessons from the use of design in global health, to distill and demystify the design methodology, and simultaneously open the conversation to perspectives and questions that can generate change and new ideas to tackle the health crises of today and those on the horizon.

## BUILDING A FOUNDATION

In the first 3 articles of this GHSP supplement, authors seek to lay a foundational understanding of design in global health.^7^^–^^9^ The authors investigate multiple vital lenses of the field, including the definition of design, the foundation and integration of design into longstanding global health practices, and the potential for improved interdisciplinary collaboration. They pose questions such as: How do you clearly define an often misunderstood field? How do design and traditional global health practices better integrate to drive more people-centered and innovative solutions to health challenges? And how do we incorporate these solutions into our other practices? These authors help us better understand what design is and how it can play a role in achieving our common health sector and global ecosystem goals.

### Defining the Field

Mishra and Sandhu^7^ give readers a perspective of what design is, show how it compares to other methodologies (including its value-add), and recommend a path forward where design is recognized as one of many essential approaches. Mishra and Sandhu^7^ contend that design has increasingly gained recognition as an effective methodology to respond better to users’ needs and wants. To global health practitioners accustomed to a more structured scientific process focused on testing hypotheses, the rapidly iterative nature of testing solutions directly with end users may make design feel arbitrary, uncomfortable, and unscientific. But the inherent tension between a structured scientific methodology and design methodology, as well as the collaborative design process—when used together—can create more sustainable and equitable outcomes. These outcomes can inform how a product or service can be best designed and introduced so that it fits within the larger system that we all live in. In this way, design is better able to take into consideration the cultural and societal norms that impact all of our behavior and decision making.

When design first burst upon the global health scene, some design practitioners may have expressed overexuberance regarding its ability to solve entrenched problems of global health. Some design practitioners likely came into the global health setting with a less than thorough understanding of the global health field. Many came in overgeneralizing design’s value by calling for a stronger emphasis on empathy. Yet, this overlooked the fact that many global health practitioners, if not the majority, had spent their careers empathizing with the struggles facing those populations for whom interventions had been designed. To overcome this potential misunderstanding, Andrawes et al.^9^ extend:

*an invitation to both designers and public health professionals to join forces more openly and more often to bring together the plurality of expertise within public health and the practical, people-centered, problem-solving approaches of design.*

### Creating a Framework

LaFond and Cherney’s^8^ work to build a shared theory of change for human-centered design (HCD) outlines potential pathways of design’s influence, articulating how HCD can

*… strengthen existing processes and introduce new processes for problem framing and solution generation and implementation by working in concert with stakeholders …*

They demonstrate the related conditions of cause and effect, beginning with the influence of design on global health programming processes and interventions and ending with the specific ways in which design can help create the “preconditions” necessary for achieving the goals of the global health ecosystem. Recognizing that the field of HCD is still in the process of generating the evidence necessary to more fully illustrate the theory of change pathways, they offer this work as an invitation to the field to use, experiment with, and evolve this shared framework.

### Integrating the Solution

As our collaboration has matured, the Design for Health community is beginning to see how the productive tensions that Andrawes et al.^9^ delineate—integrating explicit and implicit knowledge, challenging linearity with iteration, and enabling collective ownership of processes and solutions —can be a productive way forward for design and global health to come together. To harness the power of collaboration, design practitioners need global health colleagues to act as guides to understand the complex political and regulatory environments in which the field operates. Global health practitioners need the ability of design to hold and make productive the inherent tensions that can result from an iterative process that draws on multiple perspectives and areas of expertise. The examples presented in the commentary illustrate how design can provide a sound scaffolding in interdisciplinary teams and build a helpful environment for more voices to be heard and considered. Design’s generative nature enables a unique form of knowing and reasoning for problem solving and tackling complex sociocultural challenges.

## DESIGN IN ACTION

The field action reports in this issue demonstrate that design relies upon rich user engagement and an iterative approach to help adapt solutions to different cultures, as well as national, regional, and local contexts while also democratizing and strengthening the practice of global health. Not all design projects are the same. Here, we highlight what design looks like when playing the role of “spark,” as one “ingredient” in a larger whole, and when coming in as the foundation for a project “end-to-end.”^10^

### Design as Spark

The Tijani et al.^11^ article, an example of “design as spark,” demonstrates how successful co-creation approaches can lead to successful outcomes. They discuss an outbreak management system at the Nigerian Institute of Medical Research drive-through center that seeks to improve data management within the country’s current health information system. The authors propose that continued engagement of the relevant stakeholders throughout the process contributed to increased equity, sustainability, and long-term impact. The success of the system at the Institute’s drive-through center also inspired the automation of processes for other test centers around Nigeria using a similar design process. Their report demonstrates how design methods can be applied as a light touch at the beginning or middle of a project to encourage new thinking, generate new concepts, or deliver a specific output as part of a larger program.

### Design as Ingredient

Bruns^12^ discusses how HCD was an ingredient throughout a mixed-methods project by focusing on local advisory group members in South Africa as a key audience, making them integral to the intervention’s prototype successes from beginning to end. The project engaged members to ensure the prototypes/pilots that emerged were sustainable and desirable from the perspectives of both advisory group members and men living with HIV.^12^ An advisory group provided valuable input at strategic points to allow changes in direction in real-time.

Bruns proposes that the design co-creation approach, focused on the untested and untreated “last mile men” with respect to the project's problem solving, enabled an empathy for at-risk men and their caregivers across the advisory group. This became a key HCD ingredient throughout development and piloting. This empathy allowed prototypes to be rapidly evaluated because the feasibility, viability, and desirability of all parties had been considered from the beginning. This case study exemplifies “design as ingredient” as the program used parts of the design process in conjunction with other approaches from the social and behavioral sciences such as quantitative and qualitative formative research, segmentation, and ethnography.

### End-to-End Approach

Employing successful co-creation is exemplified in CyberRwanda,^13^ which was led by a multidisciplinary team of designers, public health experts, and evaluation specialists. From 2016–2019, the project deployed a youth-driven and youth-led design process with more than 600 Rwandan youth, caregivers, teachers, health care providers, and government stakeholders. CyberRwanda seeks to improve adolescent sexual and reproductive health outcomes through behavior change stories delivered via webcomics, a robust frequently-asked questions library, online ordering of health products, and a pharmacy/health facility locator, all of which provide integrated age-appropriate adolescent health and economic empowerment information and linkages to quality youth-friendly services. The Ippoliti et al. article^13^ exemplifies what the Design for Health community terms “end-to-end.” Using design in an end-to-end approach requires a full adoption of HCD methods throughout the solution development process including conducting design research to reveal new user insights relevant to the challenge or need; co-designing solutions in partnership with users or key stakeholders; gathering user feedback through prototyping and testing of ideas; and continually testing, refining, and evaluating the idea throughout implementation.

### Lessons Learned

Sharing lessons learned from integrating design in large-scale programming, Blynn et al.^14^ highlight that in public health programs deploying HCD, the user (client, provider, or community) has agency in shaping more contextually appropriate solutions. They discuss how and why that is so through a reflection of 3 projects: V, an approach to empower women to increase uptake of HIV pre-exposure prophylaxis in South Africa and Zimbabwe; Adolescents 360, an effort focusing on behavior change among adolescent girls in Ethiopia; and Reimagining Technical Assistance, a design process to rethink public health technical assistance models in Nigeria and the Democratic Republic of the Congo. These projects engage users equitably from the outset as experts in a truly collaborative, participatory, and co-creation approach. Consistent use of such an approach from project conceptualization through implementation can engender “a virtuous cycle between co-creation, stakeholder buy-in, and quality of outputs.”^14^ To reap these benefits, projects integrating HCD must be scoped differently than traditional global health programs. They must also take a more inclusive approach throughout the project in contrast to the prescriptive approach in more traditional global health programming that may perpetuate the donor-recipient relationship leading to “fragmented insights and low commitment to the process and the solutions.”^14^

## MULTIPLYING PATHS TO IMPACT

While strategies for measuring and evaluating efforts to improve human health are well-established and documented, the use of measurement in design-influenced global health programming remains a largely unexplored and much-discussed frontier. As design is increasingly integrated into global health practice, designers and global health practitioners are learning as they go how to integrate measurement into design and adapt traditional monitoring and evaluation approaches to design-influenced global health projects. There are inherent tensions in the way global health and design practitioners approach measurement. In their article, Heller et al.^15^ make the case that measurement conducted during the design process can provide additional insights that help define appropriate products, services, and interventions, as well as additive learning and proof of concept that can be critical to risk reduction in investments and program implementation. The authors use 3 recent examples of design-influenced global health interventions to illustrate how these tensions can be managed: Brilliance, a line of neonatal jaundice treatment devices; Adolescents 360, an effort focusing on behavior change among adolescent girls; and Group ANC, service design improvement for delivery of antenatal care. New approaches are required to successfully manage measurement across multidisciplinary teams, but with more transparency and greater understanding, the results have the potential to benefit global health interventions overall while optimizing the influence of design in this context.

## LOOKING TO THE FUTURE

We end this GHSP supplement by looking at what could be next for design. In the final article in this supplement, Chauhan et al.^16^ discuss the use of health futures frameworks to better align incentives and strategies to improve the impact and effectiveness of global health efforts. The authors call on us to recognize that the “future is plastic” and outline the imaginary potential of design in shaping possible, plausible, and preferable futures for individuals, communities, and societies as a whole. To do so requires shifts in mindset and practice in both global health and design, with the global health sector evolving to bring greater focus to the health of ecologies over health care, and design practitioners becoming more open to new paradigms of life-centered design and speculative design.

This supplement seeks to demonstrate that the use of design is a tool that can be used to deliver impactful health interventions that center the person as a means to increase equity, access, and usability. Through examples and case studies, we seek to present a way forward for breaking through the status quo of both design and global health. We do this directly, by calling for design to be more fully integrated into the global public health discourse. And we do this indirectly, by creating a space for plural discourses of design for health. As is evidenced in this supplement, design, if done well, can place young and adult community members, service providers, and governments—across Ethiopia, Guatemala, Kenya, India, Nigeria, Tanzania, and Uganda—in the driver’s seat. Although the examples in this supplement draw heavily from programs in Africa, similar excellent human-centered design is ongoing across the world.

Yet, to achieve our global health goals, we must realize, as Mishra and Sandhu state^7^:

*In the toolbox of approaches to global health innovation, design is critical. This toolbox is an essential processes list, and design must be on the list. ...*

with design playing an accompanying role to other disciplines to strengthen collaboration as we all strive to reach global health goals. We hope that the work shared here can act as an inspiration to design and global health practitioners alike in building a shared collective vision of how design can advance and deepen health impact.

## Notes

**Peer Reviewed**
